# Plant defensin antibacterial mode of action against *Pseudomonas* species

**DOI:** 10.1186/s12866-020-01852-1

**Published:** 2020-06-19

**Authors:** Andrew E. Sathoff, Shawn Lewenza, Deborah A. Samac

**Affiliations:** 1 Department of Plant Pathology, 1991 Upper Buford Circle, University of Minnesota, St. Paul, MN 55108 USA; 2 Department of Biology, Dakota State University, 820 N Washington Ave, Madison, SD 57042 USA; 3 Department of Microbiology and Infectious Disease, 3330 Hospital Dr. N.W., University of Calgary, Calgary, AB T2N 4Z6 Canada; 4 Faculty of Science and Technology, 1 University Dr., Athabasca University, Athabasca, AB T9S 3A3 Canada; 5 USDA-ARS, Plant Science Research Unit, 1991 Upper Buford Circle, St. Paul, MN 55108 USA

**Keywords:** Plant defensin, Antibacterial, *Medicago*, *Pseudomonas*, *lux* reporter

## Abstract

**Background:**

Though many plant defensins exhibit antibacterial activity, little is known about their antibacterial mode of action (MOA). Antimicrobial peptides with a characterized MOA induce the expression of multiple bacterial outer membrane modifications, which are required for resistance to these membrane-targeting peptides. Mini-Tn*5-lux* mutant strains of *Pseudomonas aeruginosa* with Tn insertions disrupting outer membrane protective modifications were assessed for sensitivity against plant defensin peptides. These transcriptional *lux* reporter strains were also evaluated for *lux* gene expression in response to sublethal plant defensin exposure. Also, a plant pathogen, *Pseudomonas syringae* pv. *syringae* was modified through transposon mutagenesis to create mutants that are resistant to in vitro MtDef4 treatments.

**Results:**

Plant defensins displayed specific and potent antibacterial activity against strains of *P. aeruginosa*. A defensin from *Medicago truncatula*, MtDef4, induced dose-dependent gene expression of the aminoarabinose modification of LPS and surface polycation spermidine production operons. The ability for MtDef4 to damage bacterial outer membranes was also verified visually through fluorescent microscopy. Another defensin from *M. truncatula*, MtDef5, failed to induce *lux* gene expression and limited outer membrane damage was detected with fluorescent microscopy. The transposon insertion site on MtDef4 resistant *P. syringae* pv. *syringae* mutants was sequenced, and modifications of ribosomal genes were identified to contribute to enhanced resistance to plant defensin treatments.

**Conclusions:**

MtDef4 damages the outer membrane similar to polymyxin B, which stimulates antimicrobial peptide resistance mechanisms to plant defensins. MtDef5, appears to have a different antibacterial MOA. Additionally, the MtDef4 antibacterial mode of action may also involve inhibition of translation.

## Background

Plants produce a suite of antimicrobial peptides (AMPs) to defend against the extensive array of potential pathogens encountered in their environment. Plant AMPs are classified based on their structure and presence of disulfide bonds [1]. With an abundance of representatives from diverse plant species, plant defensins are among the most widespread and best characterized plant AMPs [2]. Plant defensins are cationic, cysteine-rich antimicrobial peptides that usually contain four disulfide bonds. They have a conserved three-dimensional structure, a cysteine-stabilized *aß* (CS*aß*) motif, with a concentration of positively charged amino acid residues on the *ß*2- *ß*3 loop, which is classified as the γ-core motif (GXCX_3-9_C). The γ-core motif alone has been shown to impart antimicrobial activity and mimic the activity of the corresponding full-length defensin [3]. Plant defensins are promiscuous peptides, which means that a single peptide can have multiple distinct functions [4]. Along with having antimicrobial activity, plant defensins control plant development, contribute to zinc tolerance, and act as inhibitors of digestive enzymes [5]. In crop plants, the transgenic expression of plant defensins has been used to engineer fungal and oomycete disease resistant plants. When MsDef1, a defensin from alfalfa (*Medicago sativa*), was expressed in potato, field-grown potatoes displayed resistance to *Verticillium dahliae* [6]. NaD1, a defensin from sweet tobacco (*Nicotiana alata*), provided transgenic cotton with resistance to *Fusarium oxysporum* f. sp. *vasinfectum* and *V. dahliae* throughout 3 years of field trials [7].

Though considered to be primarily antifungal, plant defensins have been shown to demonstrate antibacterial activity against both plant and vertebrate bacterial pathogens [8]. Spinach defensin (So-D2) is the most frequently cited plant defensin with antibacterial activity, and transgenic sweet orange and grapefruit trees expressing So-D2 exhibited increased resistance to the bacterial diseases, citrus greening and citrus canker, caused by *Candidatus* Liberibacter spp. and *Xanthomonas axonopodis* pv. *citri,* respectively [9]. Plant defensins also display in vitro antibacterial activity against human pathogens. For instance, J1–1, a defensin from bell pepper (*Capsicum annum*) has a minimum inhibitory concentration (MIC) value of 250 μg/mL against *Pseudomonas aeruginosa* [10]. Also, PaDef, a defensin from avocado (*Persea americana var. drymifolia*), displays antibacterial activity against *Staphylococcus aureus* [11]. Therefore, plant defensins not only appear to be a resource for improving plant immunity to bacterial diseases but also for combatting human and animal bacterial pathogens.

A major obstacle blocking the widespread usage of plant defensins as antibacterial compounds is that their antibacterial mode of action (MOA) is poorly characterized [8] although their MOA against fungal pathogens is well-described [12–14]. Recently, the antibacterial activity of a defensin from *Medicago truncatula*, MtDef5, was characterized [15]. MtDef5 is a bi-domain defensin with two defensin domains (MtDef5A and MtDef5B) connected by a 7-amino acid linker peptide. The cationic amino acid residues found in both γ-core motifs of MtDef5 were mutated and discovered to be essential for antibacterial activity, which were the same residues previously found to be essential for antifungal activity [16]. Additionally, MtDef5 was shown to permeabilize the plasma membrane of *Xanthomonas campestris* pv. *campestris*, a gram-negative bacterial plant pathogen, but not the gram-positive plant pathogen *Clavibacter michiganensis* subsp. *nebraskensis* [15]. The MtDef5 peptide binds to DNA indicating that it may kill bacterial cells by inhibiting DNA synthesis or transcription.

The MOA of human and invertebrate defensins against bacterial pathogens is well characterized [17, 18]. Vertebrate defensins interact with the negatively charged lipopolysaccharide (LPS) in the bacterial outer membrane, which leads to swift permeabilization through pore formation [19]. For instance, HNP-1, the most investigated human α-defensin, has an antibacterial MOA typical of many AMPs. HNP-1 dimerization occurs, and through electrostatic interactions of dimers with the bacterial membrane, β-sheet dimers cross the membrane forming a pore with higher order oligomers of HNP-1 forming upon the dimers when HNP-1 is in high concentration [20]. Human β-defensin-3 (HBD3) has another well-studied antibacterial MOA. HBD3 inhibits bacterial cell wall biosynthesis through interactions with lipid II components, which enables HBD3 to have broad-spectrum antibacterial activity against both gram-positive and gram-negative bacterial species [21].

In response to the electrostatic interactions between cationic AMPs and negatively charged bacterial membranes, gram-positive and gram-negative bacteria have demonstrated the ability to modify their membrane surfaces [22]. In *P. aeruginosa* and many other gram-negative bacteria, the PhoPQ/PmrAB systems control various genes required for resistance to AMPs [23]. The *pmr* operon (*PA3552-PA3559*) is controlled by both PhoPQ and PmrAB and is required for the addition of aminoarabinose to mask the phosphates of lipid A in *P. aeruginosa* [24]. Upstream of PmrAB, the spermidine synthesis genes *PA4773* (*speD2*) and *PA4774* (*speE2*) in *P. aeruginosa* are required for production of this polycation on the outer surface of the bacterial membrane [25]. These surface modifications protect bacteria from cationic AMPs through masking of the negative surface charges, which limits AMP binding to bacterial membranes [24, 25]. The mini-Tn*5*-*luxCDABE* mutant library in *P. aeruginosa* has been used extensively to identify antimicrobial peptide MOAs and bacterial resistance mechanisms [26].

*Pseudomonas syringae* pv. *syringae* is a bacterial plant pathogen that causes bacterial stem blight of alfalfa, which is an economically important disease with widespread distribution in the Western United States [27]. Currently, there are no effective means to control bacterial stem blight of alfalfa. *P. syringae* pv. *syringae* strain ALF3, pathogenic on alfalfa and *M. truncatula*, has a draft genome sequence [28] and was shown to be sensitive to *M. truncatula* defensins, MtDef5 and MtDef4, with IC_50_ values of 0.1 and 0.4 μM, respectively [3]. Additionally, MtDef4 displays activity against *Xanthomonas alfalfae* subsp. *alfalfae* and the gram-positive bacterium *Clavibacter insidiosus*, while MtDef5 displays no activity against these pathogens [3]. There is insufficient knowledge to explain this observed specificity of plant defensin antibacterial activity.

In this study, we investigated plant defensin MOA against plant and vertebrate bacterial pathogens belonging to the genus *Pseudomonas*. Characterized *P. aeruginosa lux*-reporter strains with mutations in genes involved with cationic antimicrobial peptide resistance mechanisms were screened for sensitivity to γ-core motif plant defensin peptides. Transposon insertion libraries of *P. syringae* pv. *syringae* were generated and screened for plant defensin resistance. Generating tools to explore plant defensin MOA against bacterial plant pathogens is necessary for evaluating the risk of bacterial evolution towards defensin resistance and for the development of plant defensins into a spray-on peptide-based biological pesticide or transgenic expression of defensins for plant protection. Furthermore, knowing the antibacterial MOA of plant defensins will enhance their usage as antibacterial compounds and allow for prediction of antibacterial activity without extensive in vitro testing.

## Results

### Plant defensin derived inhibition of *Pseudomonas aeruginosa* growth

The antibacterial activity of γ-core motif peptides from MtDef4, MtDef5A, and So-D2 (Table 1) were evaluated against wild-type and antimicrobial peptide sensitive mutants of *P. aeruginosa* (Table 2). The *P. aeruginosa lux*-reporter strains had inactivated LPS modification genes, either an interrupted outer membrane surface spermidine synthesis gene (*PA4774*) or an interrupted lipid A aminoarabinose modification gene (*PA3553*). These mutants are incapable of producing outer membrane surface modifications used for protection against cationic antimicrobial peptide treatments [26, 30, 31]. Using a spread-plate assay, the *y*-core motif peptides exhibited antibacterial activity at micromolar concentrations. Against *P. aeruginosa* PAO1, the *y*-core peptides inhibited bacterial growth with MtDef4 displaying the greatest activity corresponding to an IC_50_ value of 4.2 μM (Table 3). The *lux*-reporter *P. aeruginosa* strains had the expected increase in sensitivity towards both MtDef4 and So-D2 peptides compared to the wild type strain (Table 3). Overall, MtDef5 displayed the least antibacterial activity of the evaluated *y*-core motif defensin peptides with the highest recorded IC_50_ value of 14.6 μM against *PA4774::lux*. In contrast, MtDef4 was the most potent against *PA4774::lux* with an IC_50_ value of 1.7 μM.

**Table 1:** Amino acid sequences of γ-core motif (bold) and C-terminal region (italics) of plant defensins tested in vitro

Plant Species	Defensin	Amino Acid Sequence
*Medicago truncatula*	MtDef4	**GRCRGFRRRC***FCTTHC*
*M. truncatula*	MtDef5A	**GACHRQGFGFAC***FCYKKC*
*Spinacia oleracea*	So-D2	**GDCKGIRRRC***MCSKPL*

**Table 2:** Bacterial strains used in this study

Strain or Mutant	Description	Reference
PAO1	Wild-type *Pseudomonas aeruginosa*	[29]
*PA3553::lux*	Transposon mutants and transcriptional fusion, homolog to *pmr* gene (*pmrF*) responsible for the addition of aminoarabinose to lipid A	[26]
*PA4774::lux*	Transposon mutant and transcriptional fusion, homolog to *speE* gene responsible for spermidine synthesis	[26]
ALF3	Wild-type *Pseudomonas syringae* pv. *syringae*	[28]
*ALF3::Tn5–1*	ALF3 with random transposon insertion conferring MtDef4 insensitivity, Mu_4–1	This paper
*ALF3::Tn5–2*	ALF3 with random transposon insertion conferring MtDef4 insensitivity, Mu_5–1	This paper

**Table 3:** Activity of the γ-core motif defensin peptides against *Pseudomonas aeruginosa* strains^a^

***Pseudomonas aeruginosa*** strains	MtDef4 core	MtDef5A core	So-D2 core
PAO1	4.2 ± 0.4	11.8 ± 1.4	11.6 ± 0.6
*PA3553:lux*	2.7 ± 0.3	8.5 ± 0.8	3.0 ± 0.3
*PA4774:lux*	1.7 ± 0.2	14.6 ± 1.0	5.2 ± 0.5

^a^The mean IC_50_ (μM) values are reported ± SE of three independent experiments (*n* = 3)

### Antimicrobial peptide resistance operons are induced by the MtDef4 γ-core motif peptide

**Fig. 1:**
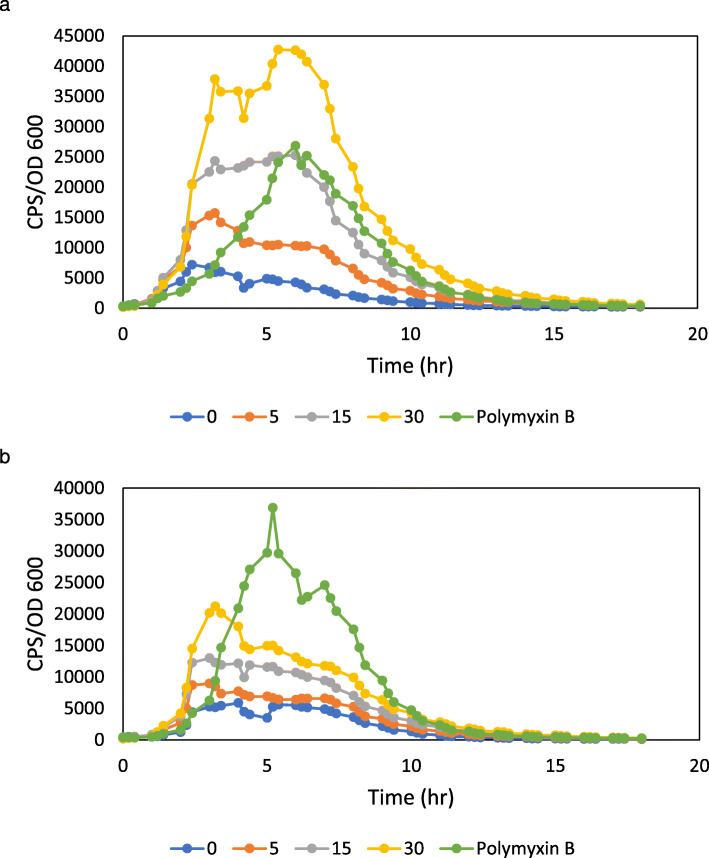
MtDef4 γ-core motif peptide induces *PA4774* and early *PA3553* gene expression. Effects of MtDef4 γ-core peptide at sub-minimal inhibitory concentrations of 0, 5, 15, or 30 μg/mL or polymyxin B at 0.5 μg/mL on the expression of the *PA4774::lux***(a)** and *PA3553::lux***(b)** transcriptional fusion in planktonic cultures in LB broth. Gene expression was normalized for growth and counts per second (CPS)/OD600 values for the average of the triplicates are presented. Each growth experiment was performed three times and representative curves are shown. The standard errors were within 10% of the mean

**Fig. 2:**
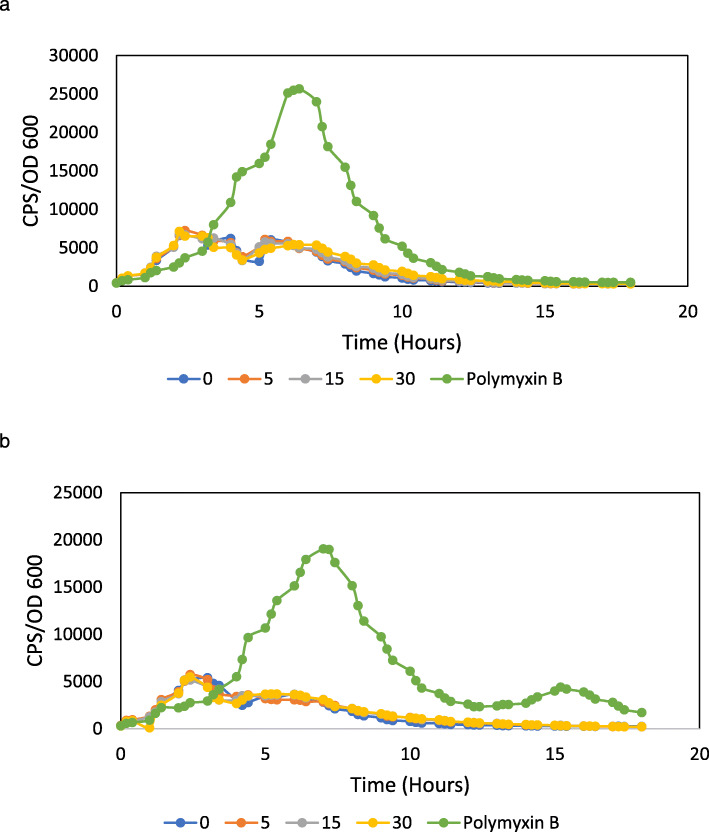
So-D2 and MtDef5 γ-core motif peptides fail to induce *PA4774* gene expression. Effects of So-D2 γ-core peptide **(a)** and MtDef5 γ-core peptide **(b)** at sub-minimal inhibitory concentrations of 0, 5, 15, or 30 μg/mL or polymyxin B at 0.5 μg/mL on the expression of the *PA4774::lux* transcriptional fusion in planktonic cultures in LB broth. Gene expression was normalized for growth and counts per second (CPS)/OD600 values for the average of the triplicates are presented. Each growth experiment was performed three times and representative curves are shown. The standard errors were within 10% of the mean

Transcriptional *lux* reporters of the *P. aeruginosa pmr* operon (*PA3552-PA3559*) and spermidine synthesis genes *speD2E2* (*PA4773-PA4774*) have been previously shown to be induced by a Mg^2+^ limiting environment, acidic pH, the presence of extracellular DNA, or the presence of antimicrobial peptides at a sublethal concentration [23, 30, 31]. We used these lux reporters under non-inducing conditions (diluted LB) to determine if exposure of these reporters to sub-MIC concentrations of plant defensins causes induced expression. The *lux-*reporter strains of *P. aeruginosa* were grown overnight in diluted LB broth, treated with plant defensin γ-core motif peptides, and monitored for bioluminescence in a microplate reader, where bioluminescence would indicate the induction of the inactivated bacterial membrane modification genes. Therefore, if the γ-core peptides cause bacterial membrane stress, the *lux*-reporter will be induced and bioluminescence will be recorded. In response to MtDef4 treatment at sublethal concentrations, *lux* expression was greatly induced in a concentration dependent manner in *PA4774::lux* compared to *PA3553*::*lux* (Fig. 1). For *PA4774*::*lux*, the level of induction from a treatment of 30 μg/mL of MtDef4 was greater than the induction caused by the antibiotic positive control, polymyxin B (0.5 μg/mL). Additionally, *PA3553::lux* expression was induced by MtDef4 at levels near those achieved by polymyxin B. MtDef5 and So-D2 failed to induce *lux* expression at levels near or greater than the antibiotic control in all mutant strains evaluated (Fig. 2). However, during the first 3 h after defensin treatment, the level of induced *lux* expression caused by all plant defensin treatments was greater than the antibiotic control, which indicates different kinetics and possibly MOAs between plant defensins and polymyxin B (Figs. 1 and 2).

### LIVE/DEAD *Bac*Light staining of defensin treated *Pseudomonas aeruginosa*

The LIVE/DEAD *Bac*Light kit consists of two fluorescent nucleic acid stains: green-fluorescent SYTO 9 stain and red-fluorescent propidium iodine stain. SYTO 9 labels bacterial cells with both intact and damaged membranes, but propidium iodine can only penetrate and label bacteria with damaged membranes. Therefore, bacteria with intact membranes will fluoresce green, while bacteria with damaged membranes will fluoresce red [32]. Both *PA4774::lux* and *PA3553::lux* were treated with either the MtDef4 or MtDef5 γ-core motif peptide and were stained with the *Bac*Light kit. MtDef4 appeared to damage the bacterial membranes of both strains, especially *PA4774::lux* (Table 4, Fig. S1). MtDef5 seemed to cause limited bacterial outer membrane damage compared to MtDef4 (Table 4, Fig. S2).

**Table 4:** Membrane permeating activity of the γ-core motif defensin peptides against *Pseudomonas aeruginosa* strains^a^

Treatment	***PA3553:lux***		***PA4774:lux***	
	% Live^**b**^	% Dead^**c**^	% Live	% Dead
MtDef4 core	71.6 ± 5.4	28.4 ± 2.5	49.4 ± 3.8	50.8 ± 1.3
MtDef5A core	99.0 ± 0.8	0.9 ± 0.3	95.4 ± 1.7	4.6 ± 2.1

^a^The mean percentage of colonies are reported ± SE of three independent experiments (*n* = 3)

^b^Live cells have intact cell membranes and were stained fluorescent green

^c^Dead cells are permeable to propidium iodine and were stained fluorescent red, which indicates membrane disruption

### *Pseudomonas syringae* pv. *syringae* transposon mutagenesis

The prior application of the mini-Tn*5*-*luxCDABE* mutant library in *P. aeruginosa* lead us to develop transposon-insertion mutant screen of a bacterial plant pathogen, *P. syringae* pv. *syringae* strain ALF3. The γ-core motif peptide of MtDef4 was previously shown to have an IC_50_ value of 3.4 μM against *P. syringae* pv. *syringae* [3], so the transposon-insertion mutants were screened for resistance at 40 μM MtDef4 (10 x IC_50_). Out of thousands of plated colonies, three slow-growing, MtDef4 resistant *P. syringae* pv. *syringae* mutants were recovered following two repetitions of the γ-core defensin peptide treatment. Genomic DNA was extracted, digested with *Eco*RI, and self-ligated with T4 DNA ligase to generate plasmids that were transformed into *E. coli*. Plasmid DNA surrounding the transposon insertion sites was sequenced for two mutants. Although sequencing was attempted from several clones of the third mutant, no sequence was obtained suggesting that the mutation was not due to a transposon insertion. The resulting sequence data from the two MtDef4 insensitive mutants (*ALF3::Tn5–1* and *ALF3::Tn5–2*) were characterized using BLAST analyses. The transposon insertion sites for both *ALF3::Tn5–1* and *ALF3::Tn5–2* were found to be located on scaffold 32544_1.1 of the ALF3 genome assembly and are 3824 base pairs apart. The mutated gene (RS24240) in *ALF3::Tn5–1* is annotated as a 16S ribosomal RNA gene, and the mutated gene (RS24220) in *ALF3::Tn5–2* is annotated as a 23S ribosomal RNA gene (Table 5).

**Table 5:** BLASTn results from the Pseudomonas Genome Database identifying the transposon insertion site in the *Pseudomonas syringae* pv. *syringae* ALF3 Tn*5* mutant strains

***Pseudomonas syringae*** pv. ***syringae*** ALF3 mutant strain	Transposon insertion site	Interrupted Gene
*ALF3::Tn5–1*	1346 in scaffold 32544_1.1	16S ribosomal RNA gene (RS24240)
*ALF3::Tn5–2*	5170 in scaffold 32544_1.1	23S ribosomal RNA gene (RS24220)

## Discussion

Plant defensins are able to kill a broad spectrum of gram-positive and gram-negative bacteria, and therefore, they are valuable candidates for generating a new class of antibiotics to treat multidrug-resistant bacteria. Full-length defensin peptides have IC_50_ values approximately ten-fold lower than those from the corresponding γ-core motif peptides [3], which indicates that full-length defensins may have a more nuanced MOA where another undiscovered motif may be acting in synergy with the γ-core. A major drawback of peptide-based antibiotics is that they are much more expensive to produce than conventional antibiotics, so to reduce cost, the size of the peptide should be minimized [33]. Truncated plant defensins (γ-core motif peptides) were assessed to evaluate a more realistic peptide-based antibacterial treatment. The plant defensin γ-core motif peptides demonstrated potent activity against *P. aeruginosa* (Table 3).

Gram-negative bacteria contain an outer membrane composed of LPS in the outer leaflet. Divalent inorganic cations (Mg^2+^ and Ca^2+^) stabilize the outer leaflet by binding neighboring LPS molecules, and the displacement of these cations by antimicrobial peptides results in membrane destabilization and bacterial cell death [34]. Polycation spermidine production and aminoarabinose-modification of lipid A contribute to reduce outer membrane permeability and therefore, the entrance of cationic AMPs [25, 35]. Random mini-Tn*5* transposon mutagenesis has been performed on *P. aeruginosa* PAO1, and the sites flanking the insertion have been sequenced and mapped, which has allowed for the characterization of outer membrane modification mutants [26]. These *P. aeruginosa* membrane modification mutants have increased sensitivity to MtDef4 and So-D2 *y*-core motif peptides with IC_50_ values reduced by 2–4 fold compared to PAO1 (Table 3). This suggests that these plant defensins may have a MOA that involves pore creation in which the displacement of divalent metal cations causes destabilization of the LPS and disruption of membrane integrity. When evaluated against the MtDef5A *y*-core motif peptide, *PA3553::lux* shows a limited increase in susceptibility and *PA4774::lux* has a modest increased resistance. This lack of greatly enhanced susceptibility implies that MtDef5 does not directly act on the bacterial outer membrane and may have an intracellular target considering that MtDef5 does not induce gene expression of the reporters. Accordingly, the fluorescent microscopy images revealed that MtDef5 caused limited outer membrane damage (Fig. S2). MtDef5 was previously shown to be internalized in *X. campestris* pv. *campestris* [15]. Also, MtDef5 demonstrates no activity towards gram-positive pathogens, *C. insidiosus* and *C. nebraskensis*, while MtDef4 had high antibacterial activity against *C. insidiosus* [3, 15]. This could be due to the inability of MtDef5 to enter the bacterial cell through the thick outer layer of peptidoglycan present in the cell wall of gram-positive cells and interact with its intracellular target. These results suggest differing MOAs between MtDef5 and the other plant defensins evaluated.

The *P. aeruginosa* mini-Tn*5*-*luxCDABE* mutants contain a promoterless luciferase gene cassette, which serves as a sensitive, real-time reporter of gene expression for the inactivated gene [26]. Highly induced expression of the *lux* gene following plant defensin treatments at sublethal concentrations signals that the defensin peptide acts on the bacterial membrane, similar to other known antimicrobial peptides [23]. MtDef4 *y*-core motif peptide treatments cause a strong concentration-dependent induction of *lux* in the *P. aeruginosa* mutant, *PA4774::lux* (Fig. 1). *PA4774::lux* also displayed the greatest level of bacterial outer membrane damage following MtDef4 treatment (Table 4, Fig. S1). The *PA4774::lux* mutant is deficient in production of outer membrane spermidine, a polyamine, which serves as a substitute for inorganic cations that bind to and stabilize LPS in the outer membrane [36]. Antimicrobial peptides compete with cations for binding to LPS, but spermidine protects the outer membrane by ensuring that the negative surface charges are masked [25]. High concentrations of exogenous spermidine (20 mM) have been demonstrated to increase the resistance of *P. aeruginosa* to cationic peptides [37]. Therefore, bacteria with high production levels of spermidine or other polyamines may be more resistant to plant defensin treatments.

The emergence of multidrug-resistant (MDR) gram-negative bacterial isolates has led to the renewed usage of both polymyxin B and colistin (polymyxin E) as therapeutic agents [38]. Polymyxins have a polycationic ring that binds to the LPS on the outer bacterial membrane and competitively displaces Ca^2+^ and Mg^2+^ leading to membrane destabilization and increased permeability [39]. With the increased prevalence of polymyxin treatments, polymyxin-resistant *P. aeruginosa* isolates have been reported worldwide [40, 41]. Throughout our study, a polymyxin B treatment was used as positive control against *P. aeruginosa*. In all *lux*-reporter assays, the plant defensin treatments displayed rapid levels of *lux* induction, and *lux*-expression was induced faster with plant defensin treatments compared to polymyxin B treatments (Fig. 1). These different induction dynamics in the *lux-*reporter assays suggest that plant defensins and polymyxin B have different MOAs on the outer membrane. Therapeutic compounds with novel MOAs are needed to treat MDR bacterial isolates, and plant defensins may be an untapped reservoir.

The transposon insertion mutants of *P. syringae* pv. *syringae*, *ALF3::Tn5–1* and *ALF3::Tn5–2*, had increased resistance to MtDef4 γ-core motif peptide treatments, which may be due to amino acid synthesis mutations or changes in ribosome structure (Table 5). The ribosome is a common target for antibacterial compounds because the alteration of bacterial ribosomes causes disruption of translation [42]. For example, aminoglycoside antibiotics target 16S rRNA in the small ribosomal subunit and tylosin targets 23S rRNA in the large ribosomal subunit [43, 44]. Target site mutations are a frequently utilized bacterial resistance mechanism. To gain resistance to several antibiotics, *Mycobacterium tuberculosis* acquired mutations in multiple regions of the *rrs* gene, which encodes 16S rRNA [45]. But, the multiplicity of rRNA genes in most bacterial species slows the development of this type of resistance [46]. Also, the antifungal MOAs of MtDef4 against *Fusarium graminearum* and *Neurospora crassa* requires *y*-core motif mediated entry into fungal cells, which implies that MtDef4 has an intracellular target [47].

Our results suggest that the antibacterial MOA of the MtDef4 *y*-core motif peptide may involve ribosomal targeting, and the transposon insertions in *P. syringae* pv. *syringae* rRNA encoding genes could be target site mutations leading to increased MtDef4 resistance. Furthermore, spermidine interacts closely with RNA because in *E. coli* cells spermidine exists predominantly as a polyamine-RNA complex [48]. Polyamines play crucial roles as modulators of RNA structure and can induce changes in RNA structure in context-dependent manner [49]. Polyamine binding to 23S rRNA on the central loop region of domain V, a site where several antibiotics are known to bind, caused structural alterations, which is suggested to restrict spiramycin binding to the ribosome [50]. In addition to having decreased outer membrane spermidine content, *PA4774::lux* may also have a reduction of intracellular spermidine. Both spermidine and MtDef4 may normally interact with 23 and 16S rRNA, but in *PA4774::lux*, this intracellular spermidine-based protection does not occur, which leads to increased susceptibility to MtDef4. The *P. syringae* pv. *syringae* transposon insertion mutants may also disrupt the interaction between MtDef4 and rRNA, which would explain the observed resistance to MtDef4. Additionally, the antibacterial MOA of MtDef4 against different *Pseudomonas* species may not be conserved or multiple MOAs could be utilized. The AMP melittin, the main component of European honeybee (*Apis mellifera*) venom, killed bacterial cells of the plant pathogen *Xanthomonas oryzae* pv. *oryzae* using multiple MOAs including membrane permeabilization, inhibition of protein synthesis, and DNA/RNA binding [51]. Also, the antifungal MOA of MtDef4 is not conserved between ascomycete fungi, *N. crassa* and *F. graminearum* [52].

## Conclusions

In this report, we gain insights into the antibacterial MOA of plant defensins against two pseudomonads, *P. aeruginosa* and *P. syringae* pv. *syringae*. In *P. aeruginosa*, we propose that MtDef4 and So-D2 interact with the bacterial outer membrane and possibly create pores leading to bacterial cell death. MtDef5 appears to have a different antibacterial MOA where outer membrane binding is not as vital and, therefore, may have an intracellular target. This hypothesis is consistent with the reported antibacterial MOA of MtDef5 against *X. campestris* pv. *campestris* in which DNA binding by MtDef5 likely interferes with DNA synthesis and transcription [15]. Additionally, plant defensins seem to have a different MOA than polymyxin B. The *P. syringae* pv. *syringae* mutational analysis suggests that MtDef4 may also target the ribosome and interfere with bacterial translation. Resistance mechanisms that bacteria use to combat MtDef4 and other plant defensins may include increased cell membrane thickness through outer membrane spermidine synthesis or target site mutations. Plant defensin γ-core motif peptides can be utilized for the development of treatments against both plant and human bacterial pathogens and for furthering knowledge of mechanisms of antimicrobial resistance.

## Methods

### Bacterial strains and growth media

All bacterial strains utilized in this study are listed in Table 2. *Pseudomonas aeruginosa* strains were obtained from Dr. Lewenza at the University of Calgary. The *P. aeruginosa lux*-reporter strains have inactivated lipopolysaccharide (LPS) modification genes, which are bacterial genes involved in the resistance to cationic antimicrobial peptides. *PA4774::lux* has an interrupted outer membrane surface spermidine synthesis gene. *PA3553::lux* has an interrupted lipid A modification gene, which is responsible for the addition of aminoarabinose to lipid A. When the *lux*-reporter bacteria produce bioluminescence, they act as a real-time reporter for the induction of the inactivated gene [26]. PAO1 was used as the wild type strain of *P. aeruginosa*. The *P. aeruginosa* strains were cultured on Luria-Bertani (LB) agar (Difco, Sparks, MD) at 37 °C. From a glycerol stock, the sequenced bacterial strain, *Pseudomonas syringae* pv. *syringae* ALF3, originally isolated from an infected alfalfa plant near Cheyenne, WY, was cultured on nutrient broth yeast extract (NBY) agar at 30 °C [28]. ALF3 was used as the wild type strain of *P. syringae* pv. *syringae*.

### Plant defensin peptide synthesis

The γ-core motif peptides derived from plant defensins, MtDef4, MtDef5A, and So-D2 [16, 53, 54] (Table 1) were chemically synthesized and purified by HPLC (LifeTein, Somerset, NJ). Lyophilized defensin peptides were rehydrated in sterile water prior to each assay.

### Determination of plant defensin antibacterial activity against *Pseudomonas aeruginosa*

To quantify defensin antibacterial activity, a spread-plate assay was used as previously described [3]. This assay was repeated three times for each strain of *P. aeruginosa*. Lawns of *P. aeruginosa* were grown on acidic LB (pH adjusted to 5.5 with HCl) plates for 15 h at 37 °C, conditions which induce antimicrobial peptide resistance mechanisms [31]. The plates were flooded with sterile water to harvest the bacteria. Cultures were diluted with sterile water to an OD_600_ of 0.1. In microcentrifuge tubes, 200 μL of bacteria were incubated at 37 °C with shaking for 3 h with various concentrations of a γ-core motif defensin peptide (0, 2.5, 5, 10, 20, or 30 μg/mL). After the defensin peptide treatment, 10-fold serial dilutions were made, and 100 μL were plated in triplicate onto LB plates. Colony forming units (CFUs) were counted for *P. aeruginosa* after incubation for 24 h at 37 °C. Regression of the average CFUs across experimental replications versus the defensin peptide concentration was used to create a dose response curve using Microsoft Excel 2016. From these dose response curves, the IC_50_ value, the amount of γ-core motif defensin peptide needed to inhibit the growth of bacterial strains by 50%, was calculated. The IC_50_ values are presented as mean ± standard error from the three repeated experiments.

### *Lux*-reporter gene expression assay

*Lux*-reporter gene expression assays, adapted from Mulcahy et al. (2008), were performed in a high-throughput manner using 96-well microplates. *P. aeruginosa* cultures were grown overnight in acidic LB broth adjusted to a pH of 5.5. Overnight cultures were diluted by 1000 into LB broth, and 150 μL of diluted culture medium with γ-core motif defensin peptide added at a sublethal concentration (0, 5, 15, or 30 μg/mL) was added to flat clear bottom 96-well microplates (Corning, Corning, NY) and overlaid with 50 μL of mineral oil to prevent evaporation. As a positive control, the antibiotic, polymyxin B, which is known to cause high gene induction of the *lux*-reporter strains, was added at a sublethal concentration of 0.5 μg/mL. Samples were assayed in triplicate. Microplate cultures were incubated at 37 °C for 18 h in a Synergy H1 microplate reader (BioTek, Winooski, VT) with optical density (600 nm) and luminescence (counts per second [CPS]) readings taken every 20 min throughout the incubation period. Gene expression values were normalized to growth (CPS/OD_600_).

### Assessment of bacterial membrane permeability through fluorescent microscopy

The *PA4774::lux* and *PA3553::lux* strains of *P. aeruginosa* were grown overnight in acidic LB broth adjusted to a pH of 5.5. Overnight cultures were diluted by 1000 in sterile water. In microcentrifuge tubes, 150 μL of the diluted bacterial suspension was treated with 30 μg/mL of either the MtDef4 or MtDef5 γ-core peptide and incubated at 37 °C for 3 h with shaking. Defensin treated bacteria were stained using a LIVE/DEAD *Bac*Light Bacterial Viability Kit (Thermo Fisher) following the manufacturer’s instructions. On a slide with one droplet of *Bac*Light mounting oil, 5 μL of the stained bacterial suspension was observed using fluorescent microscopy.

### *Pseudomonas syringae* pv. *syringae* transposon mutagenesis

An EZ-Tn*5* < R6Kγ*ori*/KAN-2 > Tnp Transposome Kit (Lucigen, Middleton, WI) was used to generate mutants of *Pseudomonas syringae* pv. *syringae* strain ALF3 through random transposon insertions. The transposome was transformed into the ALF3 strain using the *P. syringae* pv. *syringae* electroporation protocol previously described by Scholz-Schroeder [55]. The transformed bacteria were plated onto NBY agar plates with 50 mg/L kanamycin and incubated at 25 °C for 48 h. Colonies were pooled by flooding the plates with sterile water. Bacterial cultures were diluted with sterile water to an OD_600_ of 0.1. In microcentrifuge tubes, the MtDef4 γ-core motif peptide at 80 μg/mL, approximately 10 times the IC_50_ concentration, was added to 200 μL of the transformed bacteria, and the cultures were incubated at 25 °C with shaking for 3 h. After the defensin treatment, 10-fold serial dilutions were made and 100 μL were plated in triplicate onto NBY plus kanamycin plates. Plates were grown at 25 °C overnight. Single colonies were selected, restreaked on NBY plus kanamycin plates, grown overnight at 25 °C, and the defensin treatment was repeated. From the recovered *P. syringae* pv. *syringae* mutants resistant to the MtDef4 γ-core motif peptide, genomic DNA was extracted and digested with *Eco*RI (NEB, Ipswich, MA). The DNA was self-ligated using T4 DNA ligase (NEB). Electrocompetent TransforMax EC100D *pir-*116 *E. coli* (Lucigen) were electroporated with 2 μL of the ligation mix. The transformed *E. coli* were plated on LB agar plus 50 mg/L kanamycin and grown overnight at 37 °C. Plasmid DNA was extracted using a QIAprep Spin Miniprep Kit (Qiagen, Valencia, CA). The plasmid DNA was Sanger sequenced on both sides of the transposon insertion at the University of Minnesota Genomics Center using the supplied primers from the Tnp Transposome kit, KAN-2 FP-1 (5′-ACCTACAACAAAGCTCTCATCAACC − 3′) and R6KAN-2 RP-1 (5′- CTACCCTGTGGAACACCTACATCT-3′). The resulting DNA sequences near the transposon insertion were validated using Sequencer (Gene Codes Corporation, Ann Arbor, MI). Nucleotide BLAST searches using the Pseudomonas Genome Database [56] were performed on the DNA sequences near the transposon insertion site to identify the locations in the ALF3 genome of the insertions and the corresponding interrupted genes with annotations.

## Supplementary information

**Additional file 1: Figure S1.** MtDef4 γ-core motif peptide causes membrane permeabilization in both the *PA4774::lux***(a)** and *PA3553::lux***(b)** strains of *Pseudomonas aeruginosa*. Observed fluorescence using optical filters set for SYTO 9 green-fluorescent staining (left), propidium iodine red-fluorescent staining (center), and the merged images (right). Green fluorescence reveals bacterial cells with intact membranes while red fluorescence reveals bacterial cells with damaged membranes. Scale bar is 5 μm.

**Additional file 2: Figure S2.** MtDef5 γ-core motif peptide causes limited membrane permeabilization in both the *PA4774::lux***(a)** and *PA3553::lux***(b)** strains of *Pseudomonas aeruginosa*. Observed fluorescence using optical filters set for SYTO 9 green-fluorescent staining (left), propidium iodine red-fluorescent staining (center), and the merged images (right). Green fluorescence reveals bacterial cells with intact membranes while red fluorescence reveals bacterial cells with damaged membranes. Scale bar is 5 μm.

## Data Availability

The raw data generated and analyzed during this study are available from the corresponding author on reasonable request.

## Abbreviations

AMP: Antimicrobial peptide
MsDef1: *Medicago sativa* defensin 1
NaD1: *Nicotiana alata* defensin 1
So-D2: *Spinacia oleracea* defensin 2
J1–1: *Capsicum annum* defensin 1
MIC: Minimum inhibitory concentration
PaDef: *Persea americana var. drymifolia* defensin
MOA: Mode of action
MtDef5: *Medicago truncatula* defensin 5
IC_50_: Inhibitory concentration 50%
LPS: Lipopolysaccharide
HNP-1: Human α-defensin
HBD3: Human β-defensin-3
MtDef4: *Medicago truncatula* defensin 4
MDR: Multidrug-resistant
LB: Luria-Bertani
NBY: Nutrient broth yeast extract
CFU: Colony forming units
CPS: Counts per second
