# Supplementary material for: Plant defensin antibacterial mode of action against Pseudomonas species
Source: BMC Microbiol. 2020 Jun 19;20:173. doi: 10.1186/s12866-020-01852-1 (PMC7304088; doi:10.1186/s12866-020-01852-1)
Supplement: Supplementary file 1 [file 12866_2020_1852_MOESM1_ESM.docx]

a

b

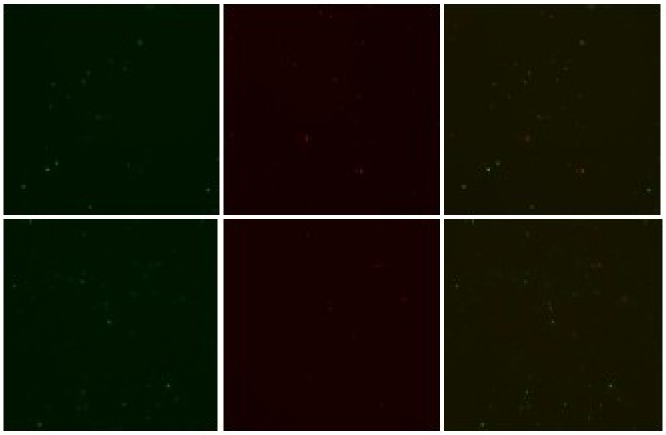

Fig. S1 MtDef4 γ-core motif peptide causes membrane permeabilization in both the *PA4774::lux* (**a**) and *PA3553::lux* (**b**) strains of *Pseudomonas aeruginosa*. Observed fluorescence using optical filters set for SYTO 9 green-fluorescent staining (left), propidium iodine red-fluorescent staining (center), and the merged images (right). Green fluorescence reveals bacterial cells with intact membranes while red fluorescence reveals bacterial cells with damaged membranes.
